# Obsessive–Compulsive Personality Disorder Symptoms in Veterans Receiving Posttraumatic Stress Disorder Specialty Care

**DOI:** 10.3390/jcm15218286

**Published:** 2026-10-24

**Authors:** Jennifer Barredo, Meghan J. Kulak, Hannah R. Swearingen, Timothy Y. Mariano, Noah S. Philip, M. Tracie Shea, Anthony Pinto, Benjamin D. Greenberg

**Affiliations:** 1 Department of Psychiatry, Warren Alpert Medical School of Brown University, Providence, RI 02906, USA; meghan_kulak@brown.edu (M.J.K.);; 2 Center for Neurorestoration and Neurotechnology, VA Providence Healthcare System, Providence, RI 02908, USA; 3 Butler Hospital, Providence, RI 02906, USA; 4 Northwell Health OCD Center, 75-59 263^rd^ Street, Glen Oaks, NY 11004, USA; 5 Department of Psychiatry, Donald and Barbara Zucker School of Medicine at Hofstra/Northwell, 270-05 76^th^ Avenue, New Hyde Park, NY 11040, USA

**Keywords:** obsessive–compulsive personality disorder, posttraumatic stress disorder, veterans, perfectionism, psychiatry

## Abstract

**Background:** Posttraumatic stress disorder (PTSD) is associated with high rates of comorbid personality disorders, which may contribute to PTSD severity. Obsessive–compulsive personality disorder (OCPD) is understudied but common among military/veterans with PTSD, with reported prevalence estimates ranging from 11 to 44%. Despite this, the relationship between OCPD traits and PTSD severity remains poorly understood. **Methods:** This retrospective chart review study examined associations between PTSD severity and OCPD traits in a naturalistic sample of 99 veterans evaluated by a single clinician in a PTSD/Trauma Recovery Services clinic. PTSD symptoms were measured with the PTSD Checklist for DSM-5 (PCL-5), and OCPD traits were measured with the Pathological Obsessive–Compulsive Personality Scale (POPS). Relationships between these two constructs were examined using Pearson’s correlations. **Results:** Overall PTSD severity was significantly and positively correlated with total OCPD traits (Spearman’s *ρ* = 0.46, *p* < 0.001). Among OCPD domains, maladaptive perfectionism showed the strongest association with PTSD severity (*ρ* = 0.45, *p* < 0.001), followed by emotional overcontrol (*ρ* = 0.45, *p* < 0.001), reluctance to delegate (*ρ* = 0.38, *p* = 0.001), rigidity (*ρ* = 0.31, *p* = 0.010), and difficulty with change (*ρ* = 0.30, *p* = 0.013). **Conclusions:** These findings suggest clinically meaningful OCPD traits were associated with PTSD symptom burden in this veteran sample, warranting further research and clinical attention.

## 1. Public Significance

Posttraumatic stress disorder (PTSD) symptoms and obsessive–compulsive personality disorder (OCPD) traits frequently co-occur and may complicate trauma treatment, but their association is relatively understudied. In this retrospective chart review of one VA clinic, higher self-reported OCPD trait burden was associated with greater concurrent self-reported PTSD symptom severity. Additional prospective studies are needed to determine whether these traits predict treatment engagement or outcomes.

## 2. Introduction

Posttraumatic stress disorder (PTSD) is a leading contributor to functional impairment and suicide risk among veterans [1]. Yet even within trauma-exposed populations, PTSD varies widely in symptom severity, functional impact, and treatment response, and the sources of this heterogeneity remain incompletely understood [2,3]. Personality factors may be one important but underexamined contributor. In particular, obsessive–compulsive personality disorder (OCPD), characterized by a pervasive need for perfection and control that constrains functioning and quality of life, may influence the emergence and persistence of trauma-related symptoms.

OCPD can be difficult to recognize because cardinal features like conscientiousness, discipline, high standards, and attention to detail may appear adaptive, especially in military and veteran contexts. What distinguishes OCPD is inflexibility, extremity, and the cost of these traits. Core features include maladaptive perfectionism, emotional overcontrol, rigidity, and rule-bound behavior [4]. OCPD is also associated with high rates of psychiatric comorbidity [5]. Because OCPD traits are often experienced as ego-syntonic [6], patients may be less likely to identify them as concerns. In clinical settings, OCPD may also receive less attention when more prominent conditions such as PTSD, depression, or anxiety disorders are present. Consequently, OCPD may remain unrecognized in some patients, and maladaptive patterns may contribute to the complexity of clinical presentations and treatment response [6]. Assessments based on either categorical or dimensional models of DSM-5 are used for clinical diagnosis. Though insufficient for formal diagnosis, additional self-report measures may be used to measure dimensional OCPD traits and may be used to screen for probable OCPD and clinical referral or to augment categorical diagnosis.

The clinical relevance of OCPD to posttraumatic stress warrants consideration because features of OCPD-like overcontrolled personality functioning align with processes observed in trauma-related distress, including difficulties with emotional regulation [7], experiential avoidance [8,9,10], and interpersonal strain [11]. For example, a strong desire for control could be associated with differences in disclosure, emotional awareness, or interpersonal connection with therapists and other supportive individuals. Maladaptive perfectionism may also be relevant to trauma-related shame and self-criticism, while rigidity may restrict openness to learning new strategies for coping with trauma reminders. Accordingly, when OCPD traits co-occur with PTSD, they may represent clinically relevant features that shape symptom presentation, interpersonal functioning, or treatment engagement.

OCPD is one of the most common personality disorders in the general population, with an estimated global prevalence rate of 6.5% [12]. It appears to be overrepresented among veterans diagnosed with PTSD, with prevalence estimates ranging from 11 to 44% [13,14,15,16]. Despite this, OCPD likely remains underdiagnosed in clinical settings. In veteran healthcare systems, diagnostic and structural disincentives may reinforce this underrecognition [17]. Hence, overcontrolled personality traits may be common among trauma-exposed veterans, yet their contribution to PTSD severity and treatment complexity remains poorly characterized.

The present naturalistic chart review extends the limited literature on OCPD in veteran populations by examining the frequency of elevated self-reported OCPD trait scores among veterans receiving trauma-related outpatient mental health care within a single psychiatrist’s clinic. We evaluated whether greater OCPD trait burden was associated with greater posttraumatic symptom severity. Characterizing this association in a real-world clinical setting may help clarify the clinical relevance of OCPD traits in trauma-focused veteran care and help identify questions for future research on the potential implications for case formulation and treatment planning.

## 3. Methods

The primary aim of this chart review was to explore associations between PTSD symptom severity and self-reported obsessive–compulsive personality traits, to provide data that may be generative for future prospective hypothesis-driven empirical work. The VA Providence Institutional Review Board approved this chart review as an exempt study for secondary data use. A waiver of informed consent was granted by the IRB. A Health Insurance Portability and Accountability Act (HIPAA) waiver was also granted, permitting retrospective review of patient assessments collected during clinical practice and treatment as usual.

### 3.1. Setting and Data Source

Clinical data were obtained from medical records of veterans seen for an initial psychiatric consult with one author (BDG), a staff psychiatrist in the Trauma Recovery Services (TRS) clinic at the Providence VA Medical Center between January 2016 and August 2025. TRS is a specialized outpatient mental health program that provides intensive treatment for veterans living with the psychological impacts of trauma. Veterans were eligible for inclusion in this chart review study if their initial consultation paperwork contained a completed Pathological Obsessive–Compulsive Personality Scale (POPS). Clinic patients completed POPS as a course of routine care during their initial consultation. Of note, no structured personality-disorder interview was administered as part of routine care. Clinical records were reviewed to obtain demographic and diagnostic information for all eligible veterans, including age, biological sex, clinical diagnoses, POPS, and PCL-5 scores. Diagnoses were extracted from patients’ comprehensive evaluation appointments and were not mutually exclusive. Initial data extraction was conducted by one author (HRS) and verified by a second (BDG).

### 3.2. Clinical Measures

#### 3.2.1. Pathological Obsessive–Compulsive Personality Scale (POPS)

The POPS [18] is a 49-item self-report measure of obsessive–compulsive personality traits and severity that includes five specific trait factors and an overall factor (total score), assessing obsessive–compulsive personality pathology severity and dysfunction. Each item is rated on a 6-point scale from 1 (*strongly disagree*) to 6 (*strongly agree*), with scores ranging between 49 and 294. The total and subscale scores have demonstrated strong internal consistency and convergent and discriminant validity with other measures of personality disorders [19]. A Receiver-Operator Characteristic (ROC) analysis conducted using POPS data obtained from a specialized OCD outpatient clinic revealed that a cut score of 178 on the POPS total correctly identified 80% of patients with OCPD, misclassifying 12% of cases [20]. Consistent with routine VA clinical practice, no structured personality-disorder interview was administered.

#### 3.2.2. PTSD Symptom Checklist for DSM-5 (PCL-5)

The PCL-5 [21] is a widely used self-report measure of PTSD. It includes 20 items that correspond to the 20 PTSD symptoms in the DSM-5, with a total score ranging from 0 to 80. PCL-5 scores have shown good internal consistency, test–retest reliability, and convergent and discriminant validity [22]. Per VHA PTSD care guidelines, scores falling between 31 and 33 are interpreted as indicative of probable PTSD.

### 3.3. Statistical Analysis

Python 3.13 with compatible pandas, numpy, scipy, and matplotlib packages was used for analyses and visualizations. Descriptive statistics (means, standard deviations, and score distributions) were calculated to characterize PTSD symptom severity (PCL-5) and obsessive–compulsive personality traits (POPS). We visually inspected score distributions using histograms and scatterplots to assess central tendency and distributional shape, with Shapiro–Wilk tests used to evaluate normality. Statistical analyses were conducted using Spearman’s partial correlations adjusting for age. All statistical tests were two-tailed with α set at 0.05. Primary analyses examined the relationship between POPS total scores and PCL total scores among veterans with available PCL-5 scores. Secondary analyses examined associations between PCL scores and each POPS subscale (Rigidity, Emotional Overcontrol, Maladaptive Perfectionism, Reluctance to Delegate, and Difficulty with Change).

## 4. Results

### 4.1. Sample Characteristics

The chart review identified 99 veterans (90.9% male) with POPS data available from their initial consultation. The most frequently diagnosed disorders were PTSD (88%), major depressive disorder (51%), obsessive–compulsive disorder (15%), and generalized anxiety disorder (7%), with other conditions diagnosed in fewer than 5% of the sample (Table 1).

Correlational analyses were limited to 68 patients with complete, continuous PTSD symptom data (PCL-5). Although 88% of the full sample carried a documented PTSD diagnosis, PCL-5 scores were unavailable for a subsample of patients referred to this clinic previously evaluated using alternative measures (e.g., Clinician-Administered PTSD Scale for *DSM-5*). To address potential bias from this restriction, patients with (*n* = 68) and without PCL-5 data (*n* = 31) were compared on available demographic characteristics. Patients with complete PCL-5 data were older (M = 50.41, SD = 15.71) than those with missing data (M = 39.74, SD = 12.19), *t*(97) = 3.35, *p* = 0.001, but did not differ in POPS total scores, *t*(97) = −0.73, *p* = 0.464, or any POPS subscale (all *p*s > 0.181). Female veterans accounted for 11.8% (*n* = 8) of patients with complete PCL-5 data, compared with 3% (*n* = 1) of those without scores. Missingness varied by referral year, concentrated in patients referred in 2018 (14 missing vs. 3 complete) and lower in other years (2016: 1 missing vs. 23 complete; 2022: 1 missing vs. 12 complete). Diagnostic profiles were heterogeneous across groups, with PTSD and PTSD/MDD profiles among the most common (Table 1). Overall, missingness was primarily associated with age and referral year. Other demographics were too infrequent to parse by group and are summarized in Table 2.

### 4.2. Frequency of Elevated OCPD Trait Scores

The mean POPS total score was 175.78 (SD = 37.57) in the full sample with available scores (*n* = 99), suggesting that OCPD symptoms were clinically meaningful, approaching the 80% sensitivity threshold (total POPS > 177) reported in a study examining comorbidity in individuals with OCD in a specialized outpatient clinic [20]. Indeed, 55/99 (55.6%) veterans met the rating scale-defined threshold for clinically significant OCPD (i.e., total POPS score > 177).

### 4.3. PTSD Symptom Severity and OCPD Traits

**Figure 1:**
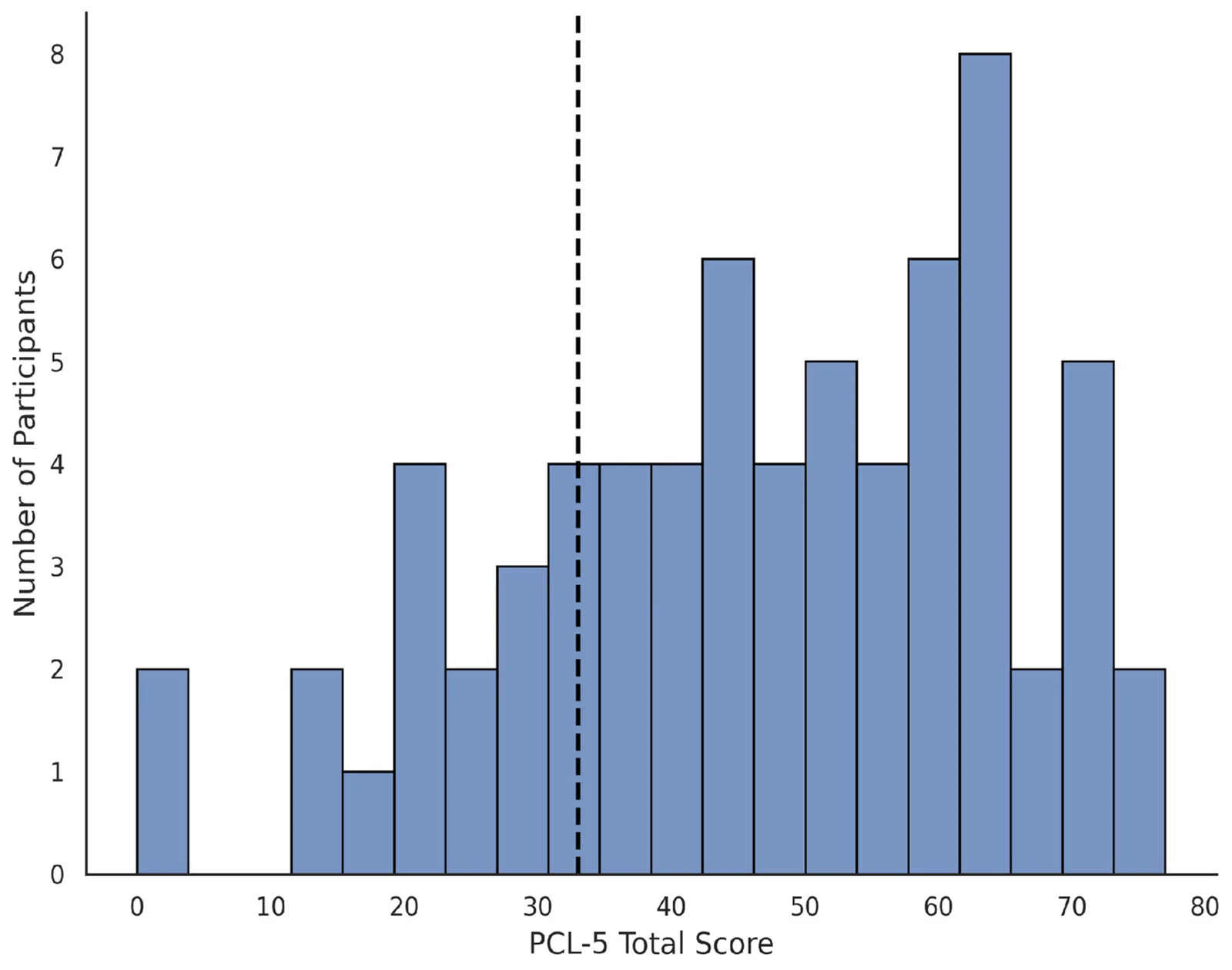
Distribution of PCL-5 total scores for 68 veterans. The dashed line corresponds to an overall score of 33, indicative of clinically significant severity and often considered suggestive of probable PTSD diagnosis.

**Figure 2:**
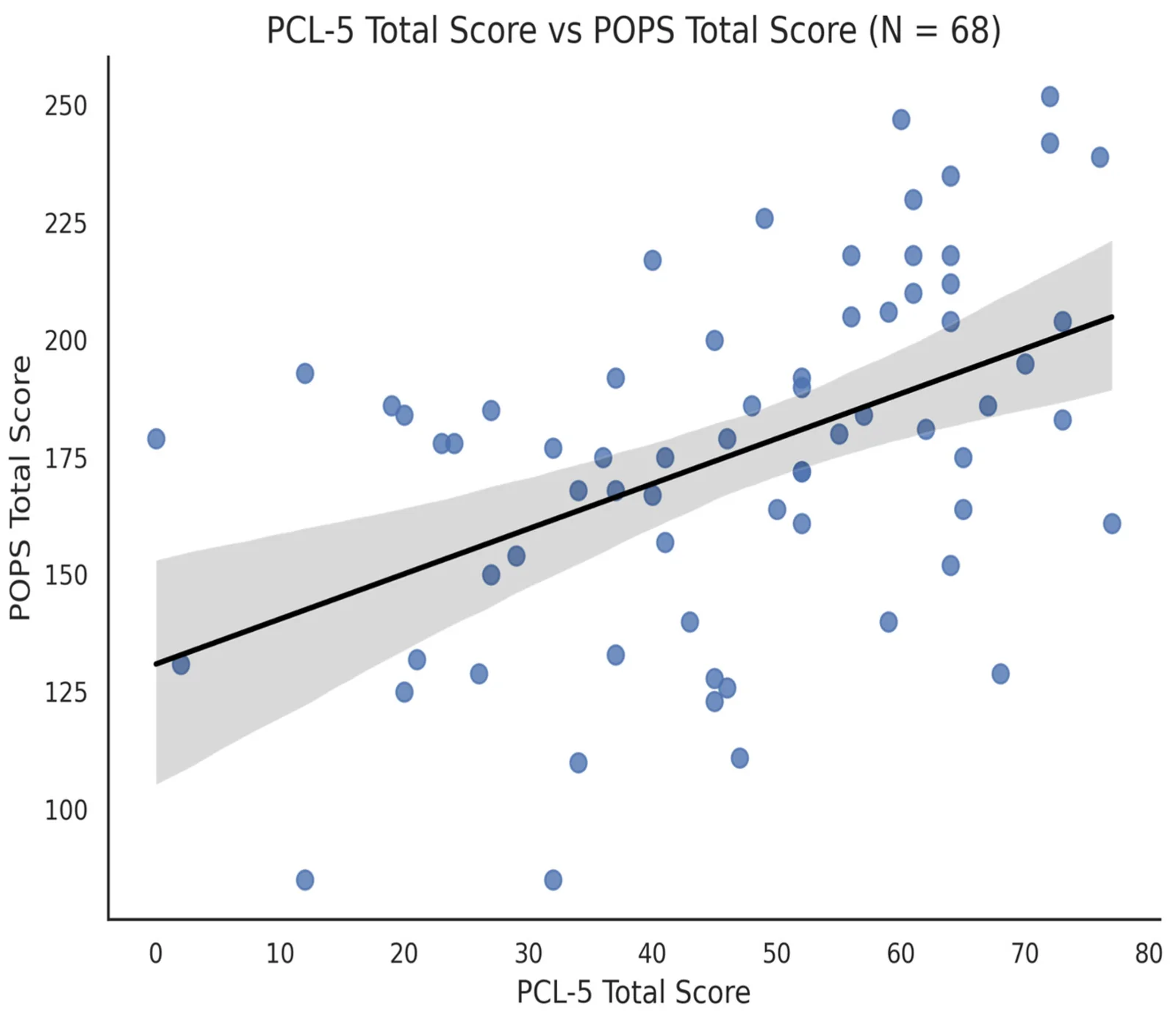
Scatterplot illustrating the relationship between PCL-5 total scores and POPS total scores. The solid line represents the fitted linear regression (Spearman’s ρ = 0.46, *p* < 0.001), with grey denoting the 95% confidence interval.

Among the 68 veterans with both POPS and PCL scores, 52.9% had an overall POPS score meeting or exceeding >177 (*n* = 36). In this subsample, the mean PCL total score was *M* = 46.65 (*SD* = 18.61) (Figure 1). POPS scores did not deviate from normality, Shapiro–Wilk W = 0.98, *p* = 0.461, while PCL-5 showed a trend toward deviation, Shapiro–Wilk W = 0.97, *p* = 0.06. In light of this trend, we report more conservative Spearman’s, rather than Pearson correlations. We observed a moderate, positive correlation between PTSD symptom severity and OCPD traits, Spearman’s *p* = 0.46, *ρ*^2^ = 0.21, 95% CI [0.25, 0.63], *p* < 0.001 (Figure 2). Partial correlations controlling for age were equivalent, *ρ* = 0.47, *ρ*^2^ = 0.22, 95% CI [0.26, 0.64], *p* < 0.001. A sensitivity analysis indicated that, with an analytic sample of 68 participants, the study had 80% power at a = 0.05 (two-sided) to detect correlations of approximately *ρ* = 0.33 or greater, with the same sensitivity to detect a two-sided partial correlation with one covariate.

### 4.4. Associations with OCPD Subscales

Spearman’s partial correlations (controlling for age) indicated that PTSD severity was positively associated with all five OCPD domains. The strongest association was observed for maladaptive perfectionism, Spearman’s *ρ* = 0.45, *ρ*^2^ = 0.20, 95% CI [0.24, 0.62], *p* < 0.001, followed by emotional overcontrol, *ρ* = 0.44, *ρ*^2^ = 0.19, 95% CI [0.22, 0.61], *p* < 0.001, reluctance to delegate, *ρ* = 0.38, *ρ*^2^ = 0.14, 95% CI [0.15, 0.56], *p* = 0.001, rigidity, *ρ* = 0.31, *ρ*^2^ = 0.10, 95% CI [0.08, 0.51], *p* = 0.010, and difficulty with change, *ρ* = 0.30, *ρ*^2^ = 0.09, 95% CI [0.06, 0.50], *p* = 0.013. Note that the Bonferroni-adjusted significant threshold for these exploratory analyses was *p* < 0.01, so associations between PCL-5 scores and the POPS rigidity and reluctance to delegate subscales should be viewed as trending.

## 5. Discussion

In this naturalistic retrospective chart review study of outpatient veterans, we found that OCPD traits were significantly associated with PTSD symptom severity. Among the POPS subscales, maladaptive perfectionism and emotional overcontrol showed the strongest associations with PTSD severity. These initial exploratory findings suggest that OCPD traits, which have received relatively limited attention in trauma research and clinical practice, may be associated with greater symptom burden among veterans receiving VA trauma-related care.

These findings may be clinically relevant because OCPD can be difficult to recognize and may be underdiagnosed, particularly in veteran populations [17]. Historically, personality disorder diagnoses have also carried implications for service-connected disability determination, which could influence diagnoses in this clinical setting. Personality-related features may be less readily identified when symptoms are primarily understood within a military/veteran trauma-related framework. Together, these factors may contribute to reduced recognition of OCPD in veterans receiving trauma-related mental healthcare.

Challenges in identifying OCPD may extend beyond documentation to the clinical recognition of OCPD itself. The clinical presentation of OCPD can be subtle, heterogeneous, and difficult to parse from aspects of trauma and posttraumatic symptoms. Prior work supports a two-factor model of OCPD comprising a controlling subtype and an anxious subtype [6,23]. The controlling subtype is characterized by rigid rules and routines, verbal hostility, anger outbursts, and efforts to impose high perfectionistic standards on oneself and others. Clinically, these features may present as inflexibility, irritability, and interpersonal conflict and may be more readily apparent in routine care. In contrast, the anxious subtype may present less overtly. Rather than presenting as domineering or hostile, these individuals often appear conscientious, conflict-avoidant, self-critical, excessively detail-focused, and prone to procrastination or difficulty completing tasks. Many patients show features of both, with different traits becoming more prominent depending on context [6].

This heterogeneity has important implications for veterans receiving trauma care. While both OCPD presentations may be maladaptive, they may manifest differently in clinical settings. When controlling aspects predominate, features such as anger, interpersonal conflict, and emotional dysregulation may be particularly salient in patients with comorbid trauma-related symptoms [6]. In contrast, the anxious subtype may present with features such as avoidance and functional impairment that may overlap with posttraumatic avoidance. Notably, the two OCPD features most strongly associated with PTSD symptom severity in this study, emotional overcontrol and maladaptive perfectionism, cut across these subtypes. A clinically important future question, then, may not be simply whether OCPD traits are present, but how they are organized within a given patient and how underlying metacognitive models align with aspects of PTSD, as has been explored in OCD [24] and eating disorders [25]. This study did not assess OCPD subtypes or trait configurations directly, although this may be an important area of future exploration.

The organization and balance of OCPD traits may be clinically relevant because different features may relate to broader mental health and treatment concerns. Emotional overcontrol has been associated with internalizing symptoms, social isolation, and reduced emotional awareness [26], while maladaptive perfectionism has been associated with shame, self-criticism, and avoidance. Patients with these features may also have greater difficulty describing their emotions, tolerating vulnerability, or evaluating treatment progress without imposing rigid or self-critical standards. These traits may also be associated with greater PTSD symptom-related emotional dysregulation, which in turn can contribute to anger and poorer social quality of life [27]. Of note, the combination of elevated perfectionism and emotional overcontrol has been associated with elevated suicide risk [26].

OCPD traits may also have implications for posttraumatic treatment, although these possibilities were not directly assessed in the present study. For example, perfectionistic or inflexible tendencies could potentially affect how patients interpret treatment instructions, respond to exposure-based interventions, or tolerate uncertainty during treatment. Similarly, emotional overcontrol could potentially affect emotional disclosure or willingness to tolerate vulnerability. These possibilities provide clinically relevant hypotheses for future research, but treatment engagement, therapeutic alliance, and treatment response were not measured in this study. Accordingly, the present findings should not be interpreted as demonstrating that OCPD traits influence PTSD treatment processes. Prospective studies examining OCPD traits alongside treatment engagement, therapeutic alliance, and treatment outcomes are needed.

Unrecognized OCPD traits can also affect clinicians. Although empirical work in this area remains limited, civilian studies have linked OCPD with therapist disengagement, boredom, annoyance, and withdrawal [28]. More broadly, negative countertransference has been linked to poorer therapeutic alliance and likelihood of treatment discontinuation across outpatient mental health clinics [29]. These dynamics are consequential in PTSD care, where clinician engagement and alliance are established predictors of outcome [30,31]. For these reasons, systematic screening for OCPD traits may be useful in some veterans receiving PTSD treatment. Brief self-report measures such as the POPS offer a practical way to identify clinically relevant traits [6]. Earlier recognition may provide additional context for case formulation and treatment planning, particularly when rigidity, perfectionism, or emotional overcontrol are prominent features of clinical presentation. Though this could not be examined in the current study, it warrants further prospective examination in veterans and treatment providers.

Treatment options for OCPD remain limited. No pharmacologic intervention is approved for OCPD by the U.S. Food and Drug Administration, though selective serotonin reuptake inhibitors may reduce maladaptive rigidity and perfectionism in some patients [32,33]. Cognitive behavioral therapy (CBT) and Acceptance and Commitment Therapy (ACT) [6,34], radically open dialectical behavioral therapy [35,36], and metacognitive therapy have shown efficacy for OCPD treatment [37]. When specialized therapy is unavailable, psychoeducation, brief CBT-based interventions, and bibliotherapy may be incorporated into routine clinical care [38]. Treatment may include identifying rigid or perfectionistic patterns and exploring the contexts in which they are maintained. Behavioral experiments can be used to examine inflexible rules and standards, while encouraging greater behavioral flexibility and self-compassion rather than self-criticism. Educational resources, including “Tightrope Walking” may also support patients and families [39]. Skills-based approaches targeting emotion regulation and flexibility may also have additional relevance to improve psychosocial functioning [40].

This study has several limitations, and the observed association between POPS and PCL-5 scores should not be interpreted as demonstrating an independent relationship between OCPD traits and PTSD symptom severity. Although sensitivity analyses adjusted for age, the practical constraints of a retrospective chart review precluded consistent examination of contemporaneous depression and anxiety symptoms. Diagnostic information extracted from the clinical record could not fully address this limitation because diagnoses were not mutually exclusive and did not quantify symptom severity at the time the POPS and PCL-5 were completed. Residual confounding by general psychiatric distress or negative affect therefore remains possible. In addition, both primary measures were self-report instruments administered in the same clinical context, introducing the possibility of common-method variance. Accordingly, these findings are best interpreted as evidence of an association requiring prospective replication using systematic measurement of relevant clinical covariates rather than evidence that OCPD traits independently predict PTSD symptom severity. As noted earlier in the manuscript, the sample was drawn from one psychiatrist’s outpatient practice within the VA medical system. Most patients in that caseload were transferred from other clinicians who had left the TRS clinic, which may have reduced but does not eliminate the potential for systematic referral bias. However, the provider’s expertise in obsessive–compulsive and related disorders may have influenced more recent referrals. The observed frequency of elevated OCPD trait scores of elevated scores on the POPS (55.6%) in this sample was nominally higher than in other studies of veterans (range: 11–44%; [13,14,15,16], but we acknowledge that self-reported POPS scores may differ from formal clinical diagnoses.

The sample was predominantly male and White, limiting generalizability across sex and race. Women comprised 9.1% of the full sample (*n* = 9) and 11.8% of the analytic subsample with available PCL-5 data (*n* = 8), precluding meaningful evaluation of sex-related differences in the observed associations. Research examining PTSD symptom severity in female trauma-exposed populations underscores the importance of studying groups whose experiences may be underrepresented in trauma research [41]. Future studies should recruit more diverse veteran samples and examine whether associations between OCPD traits and PTSD symptom severity differ across sex, race, and other sociodemographic characteristics. Trauma exposure and sociocultural context may also influence the presentation and interpretation of posttraumatic and obsessive–compulsive symptoms [42,43,44]. Because the present study did not examine cross-cultural differences in symptom assessment or interpretation, these factors warrant consideration in future research. Finally, the retrospective, cross-sectional design precludes conclusions about causality or the longitudinal impact of OCPD traits on the PTSD course and treatment outcomes.

## 6. Conclusions

In this chart review of outpatient veterans, OCPD traits were significantly associated with PTSD symptom severity, with emotional overcontrol and maladaptive perfectionism showing the strongest associations. These findings suggest that specific dimensions of OCPD may be clinically relevant to PTSD symptom burden in veterans. Subsequent studies should examine how distinct configurations of OCPD traits are associated with PTSD presentation and outcomes and whether systematic screening adds value to case formulation, treatment planning, and long-term care for trauma-exposed veterans.

## Figures and Tables

**Table 1:** Mental Health Diagnoses.

	Full Sample, *n* (%)	Analytic Sample, *n* (%)
PTSD	88 (88.89)	63 (92.65)
MDD	51 (51.52)	37 (54.41)
OCD	15 (15.15)	6 (8.82)
GAD	7 (7.07)	6 (8.82)
Bipolar	5 (5.04)	3 (4.41)
ADHD	4 (4.04)	2 (2.94)
Panic disorder	4 (4.04)	2 (2.94)
BPD	3 (3.03)	1 (1.47)
OCPD	2 (2.02)	0 (0.00)
SUD	1 (1.01)	1 (1.47)
Schizophrenia	1 (1.01)	0 (0.00)
Social Phobia	1 (1.01)	0 (0.00)

Note. Diagnoses are not mutually exclusive; patients may be represented in more than one diagnostic category. Patients with a PTSD diagnosis excluded from the analytic sample did not have a recent PCL-5 assessment (within 3 months prior to referral) available in the medical record. *MDD* = major depressive disorder; *OCD* = obsessive–compulsive disorder; *GAD* = generalized anxiety disorder; *Bipolar* = bipolar I or II disorder; *ADHD* = attention-deficit/hyperactivity disorder; *BPD* = borderline personality disorder; *SUD* = substance use disorder.

**Table 2:** Race and ethnicity.

	White	Black or African American	Asian or Pacific Islander	American Indian or Alaskan Native	Multi-Racial	Other	Unknown	Total
Hispanic	8	2	0	0	0	0	0	10
Non-Hispanic	77	5	4	0	1	0	1	88
Unknown	0	0	0	0	0	0	1	1
Total	85	7	4	0	1	0	2	99

## Data Availability

Data is available upon request of the corresponding author.
